# Infected simple renal cyst due to *Streptococcus pneumoniae* rapidly diagnosed by the melting temperature mapping method: a case report

**DOI:** 10.1186/s12887-021-02736-7

**Published:** 2021-06-05

**Authors:** Yoji Uejima, Hideki Niimi, Reiko Kato, Mihoko Furuichi, Satoshi Sato, Isao Kitajima, Yutaka Kawano, Tsutomu Oh-Ishi, Hiroshi Kawashima, Eisuke Suganuma

**Affiliations:** 1 Division of Infectious Diseases and Immunology, Saitama Children’s Medical Center, 1-2 Shintoshin, Chuou-ku, 330-8777 Saitama, Japan; 2 Department of Clinical Laboratory and Molecular Pathology, Faculty of Medicine, Academic Assembly, University of Toyama, Toyama, Japan; 3 Department of Pediatric Surgery, Saitama Children’s Medical Center, Saitama, Japan

**Keywords:** *Streptococcus pneumoniae*, infected simple renal cyst, melting temperature mapping method, rapid detection of pathogenic bacteria, antimicrobial stewardship

## Abstract

**Background:**

Spontaneous infection of preexisting solitary renal cysts has been documented in adults but is extremely rare in children. To date, no cases of simple renal cysts infected with *Streptococcus pneumoniae* have been described. Recently, reports have described the diagnosis of bacterial infection using the 16 S rRNA gene as well as the accompanying antimicrobial stewardship for microorganisms that are difficult to culture and for culture-negative cases after preceding antibacterial administration.

**Case presentation:**

A four-year-old Japanese girl who had a pleuroperitoneal shunt inserted to drain a right pleural effusion due to occlusion of the hepatic portion of the inferior vena cava at three years old visited our hospital due to fever and respiratory discomfort. She was incidentally found to have a right simple renal cyst 10 months before admission. The patient was suspected to have pneumonitis or catheter-related blood stream infection on chest X-ray, which showed right-side pleural effusion. She was diagnosed with invasive pneumococcal infection, as *Streptococcus pneumoniae* was detected from blood culture on admission. Transient improvements in her symptoms and decreases in the white blood cell count and C-reactive protein level were observed after effective antibiotic administration, but her respiratory condition deteriorated. Enhanced CT showed right renal cyst enlargement and enhancement and thickening of the surrounding wall. Using the melting temperature (Tm) mapping method, *S. pneumoniae* was rapidly detected directly from pus 4.5 hours after drainage. The specimen culture was negative, but the extracted 16 S rDNA sequence revealed 100 % identity for *S. pneumoniae* from the same specimen the subsequent day. We successfully performed optimal treatment and reduced medical cost based on the positive Tm mapping method result.

**Conclusions:**

We report the first case of a *S. pneumoniae*-infected simple renal cyst. The drainage culture was negative, but the Tm mapping method rapidly detected *S. pneumoniae* directly from the drainage. The Tm mapping method may have great impacts on rapid diagnosis and effective antimicrobial stewardship.

## Background

The incidence of simple renal cysts in children is rare, with an incidence of less than 0.5 % in children [1], and infected renal cysts are even more rare. Hematogenous spread, retrograde infection from the urinary tract or direct penetration to the simple renal cyst are thought to be the most common causes of infected renal cysts. The most common bacterial cause of infected simple renal cysts in adults is *Escherichia coli* [2].

*Streptococcus pneumoniae* is a clinically important pathogenic bacterium in childhood as a causative agent of respiratory infections, such as pneumonia, sinusitis, and otitis media, as well as serious infections, such as meningitis and sepsis [3]. However, to date, there have been no previous reports of pneumococcal infection of preexisting simple renal cysts.

We often experience that culture from clinical specimens collected after administering antibiotics is negative. Rapid and accurate identification of pathogenic bacteria from clinical specimens is crucial for the management of bacterial infections. The original “melting temperature (Tm) mapping method” for rapidly identifying the dominant bacteria in a clinical sample from a sterile site was reported by Niimi et al. [4]. This study suggested that more than 100 bacterial species can be identified by employing only seven primer sets and that these findings can be obtained within 3 h of sterile site collection. Here, we report a case of an infected simple renal cyst due to *S. pneumoniae*, which was diagnosed by the Tm mapping method.

## Case presentation

**Fig. 1:**
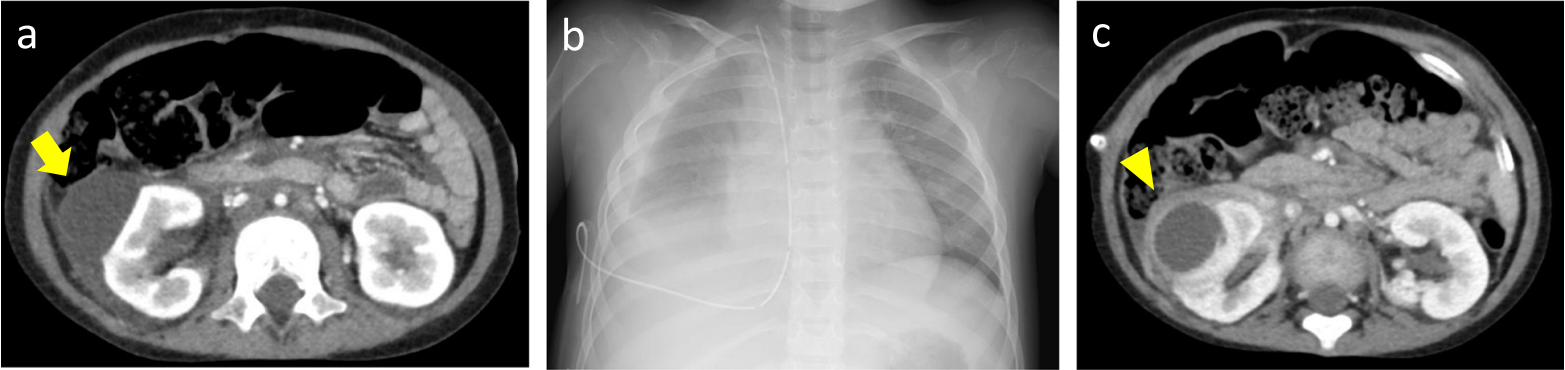
**a** Enhanced computed tomography showing a 4 cm size, nonenhanced, single, round and thin-walled cyst in the right upper kidney ten months before admission, indicating a simple renal cyst. **b** Chest X-ray showing massive pleural effusion on the right side on admission. **c** Enhanced computed tomography showing compression of the renal parenchyma, pelvis and ureter due to the enlargement of the right renal cyst and enhancement and thickening of the wall around the cyst, with perirenal inflammatory changes, on hospital day 21

A Japanese four-year-old girl with a rhinorrhea and four days of fever was referred to our hospital. Her past history was as follows: she was delivered at 35 weeks of gestation by caesarian section because of nonimmune hydrops fetalis secondary to congenital chylothorax. She was diagnosed with Sturge-Weber syndrome at 2 years old due to hemangiomas in the trigeminal nerve distribution area, glaucoma, occipital lobe hemangioma and parenchymal atrophy in the same area, and developmental disabilities. Pleural effusion was present at three years of age because of Budd-Chiari syndrome due to occlusion of the hepatic portion of the inferior vena cava. After the placement of a pleuroperitoneal shunt, pleural effusions were improved. An abdominal computed tomography (CT) incidentally revealed a simple renal cyst in her right kidney, and the asymptomatic simple renal cyst was managed conservatively (Fig. 1a). Her immunizations were up-to-date, and she received a 13-valent pneumococcal conjugate vaccine four times.

**Fig. 2:**
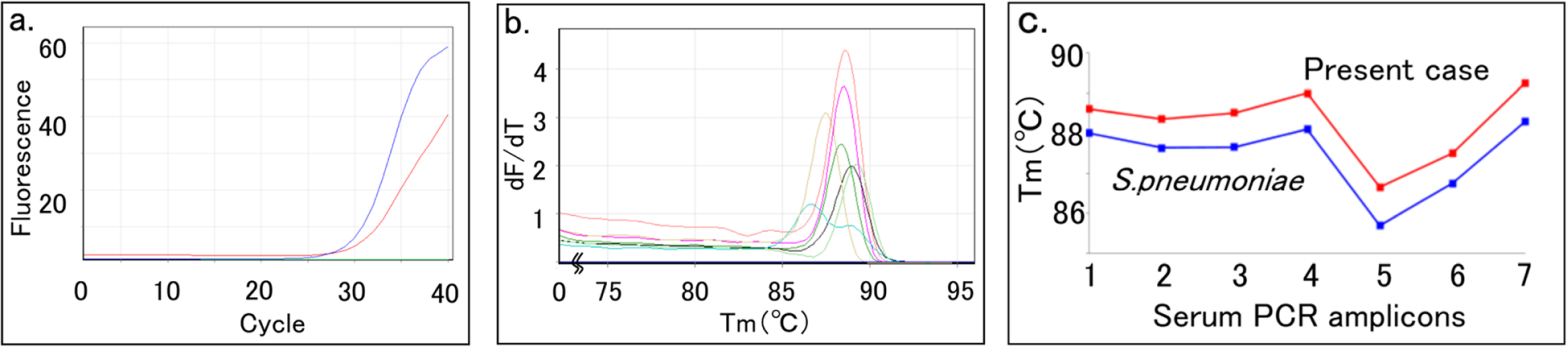
Identification of bacterial pathogens using the melting temperature mapping method. **a** Amplification of the 16 S rDNA gene extracted from pus, positive control (*Escherichia coli*) and negative control (highly pure water) indicated by the red line, blue line and green line, respectively, in the 1st PCR. **b** Melting curves of the seven amplicons in the 2nd PCR. **c** The seven melting temperatures of the amplicons were mapped, and their plot matches the plot of *Streptococcus pneumoniae* in the database

On exam, she was initially febrile: her axillary temperature was 38.1 °C; respiratory rate was 28/min, with an O_2_ saturation of 97 % on room air; heart rate was 144/min; and blood pressure was 144/108 mmHg. She had tenderness on her back, and her breath sounds were decreased. Her white blood cell (WBC) count was 15,000/µl, with differential counts of neutrophils (72.6 %) and lymphocytes (20.2 %); her C-reactive protein level was markedly elevated (28.48 mg/dl, normal range 0-0.3 mg/dl); her serum total protein level was 6.8 g/dl and her serum albumin level was slightly decreased (3.0 g/dl, normal range 3.9–5.2 mg/dl). She did not show symptoms of nephrotic syndrome, such as edema and heavy proteinuria, or symptoms of protein losing enteropathy, such as diarrhea. Pyuria and bacteriuria were not present in her urinalysis. A chest X-ray showed massive pleural effusions on the right side (Fig. 1b). Analysis of the pleural fluid obtained by thoracentesis revealed the following: pH, 7.011; protein, 900 mg/dl; and WBC, 2,940 cells/µl (66 % neutrophils). We suspected pneumococcal infection or catheter-related blood stream infection due to the presence of a pleuroperitoneal shunt catheter and initiated 200 mg/kg cefotaxime (CTX) per day and 60 mg/kg vancomycin (VCM) per day. VCM was discontinued after two days. *S. pneumoniae* was detected from two sets of blood cultures on admission, chest CT showed infiltration in the left lower lobe and right upper lobe, and otitis media was noted by otological evaluation on the subsequent day. We diagnosed her with invasive pneumococcal disease, including pneumonitis and otitis media. Based on susceptibility test results, CTX was changed to 200 mg/kg ampicillin per day on day 5. Although transient improvement of her symptoms and a decrease in the WBC count (9,800/µl) and CRP level (11.9 mg/dl) were observed on hospital day 9, she complained of respiratory discomfort again on hospital day 12. Her WBC count (13,200/µl) and serum CRP level (16.41 mg/dl) were elevated. Ampicillin (ABPC) treatment was escalated to 300 mg/kg tazobactam-piperacillin (TAZ/PIPC) treatment per day empirically. However, her symptoms did not improve sufficiently. Ultrasonography on hospital day 20 revealed a fluid-fluid level, suggesting an abscess in the right renal cyst. Enhanced CT on hospital day 21 revealed enlargement of the right renal cyst and enhancement and thickening of the wall around the cyst, which was considered to be an infected simple renal cyst (Fig. 1c). A drainage procedure under general anesthesia was performed on hospital day 23. Twelve milliliters was removed of greenish white pus was removed, and Gram staining of the pus showed neutrophils with no organisms. The melting temperature (Tm) mapping method was performed using 50 µl of the pus. After bacterial DNA was isolated from the pellets using a DNA extraction kit (DNA Extraction Kit, Mitsui Chemicals, Japan) in accordance with the supplier’s instructions, we conducted nested polymerase chain reaction (PCR) on the DNA using seven universal bacterial primer sets and EvaGreen dye, which was performed on an analytical instrument with a high degree of thermal accuracy among PCR tubes. The data profile was analyzed using a Rotor-Gene Q® (QIAGEN, Germany), which can achieve temperature uniformity among samples (± 0.02 °C), and the Tm values were identified. The seven Tm values were determined and mapped on two dimensions, and by comparing the pattern to those in the database, *S. pneumoniae* was identified 4.5 hours after cyst drainage (Fig. 2). We diagnosed her with a simple renal cyst infected with *S. pneumoniae*. The culture of the pus was negative, but we de-escalated TAZ/PIPC treatment to 200 mg/kg ABPC treatment per day on the basis of positive detection by the Tm mapping method on hospital day 27. Her convalescence was uneventful after the drainage, the drainage tube was removed on hospital day 29, and the antibiotic treatment was changed to oral amoxicillin (40 mg/kg per day) on hospital day 30. An asymptomatic scar approximately 1 cm in size was observed in her right kidney on ultrasonography, but she was discharged from our hospital with 5 days of oral amoxicillin on hospital day 32. Samples of fluid aspirated from the renal cyst did not grow any organisms in culture, but 16 S rRNA sequencing of the pus revealed a 100 % nucleotide identity to the respective sequence of a *S. pneumoniae* strain (ABI 3500 Genetic Analyzer, Applied Biosystems, Foster City, CA) in subsequent days. She was followed up for two years after discharge, and no recurrence of renal cyst infection was observed.

## Discussion and conclusions

The pathogenic mechanisms of simple renal cysts in children are still unknown. Simple renal cysts were previously reported to be rare in children, but with the development and prevalence of ultrasonography, the frequency of detection is increasing in the fetal and neonatal periods [5, 6]. Renal cysts in adults were classically classified on the basis of their characteristics by Bosniak in 1986, and the classifications were refined in 2003 [7]. The management of pediatric cystic kidney disease has been gradually improved. An international consensus statement on the diagnosis and management of autosomal dominant polycystic kidney disease (ADPKD) in children and young people was announced [8]. Imaging of kidney cysts and cystic kidney diseases in children was established by an international working group [9]. In our case, enhanced CT incidentally showed a mass in the right kidney ten months before admission. The mass was a nonenhanced, single, round and thin-walled cyst. This cyst was classified as Bosniak category I within the Bosniak classification system. In ultrasonographic findings, this cyst was round, thin-walled, anechoic, and nonseptated and had no Doppler blood flow related to the cyst, which was categorized as a simple cyst by an international working group consensus statement.

*S. pneumoniae* is generally known as a causative pathogen of diseases such as bacteremia, meningitis, pneumonia and otitis media [3]. In addition, although relatively infrequent, pneumococcal pyelonephritis, urosepsis and urinary tract infections have been reported [10, 11]. Metastatic pyogenic infections can occur during the course of bacteremia caused by invasive pneumococcal disease with and without underlying immunological disorders [12, 13]. Lantinga et al. [2] conducted a systematic review from 1948 to 2014 and reported that various pathogenic microorganisms, including bacteria and fungi, were causative agents according to microbiological results and pathogens isolated in renal cyst infections. *E. coli* was the most frequent, but *S. pneumoniae* was not detected in their study population. A review of the English-language literature found four cases in which *S. pneumoniae* was the pathogen in renal abscess in adults [10, 14–16]. However, no renal abscess or infected renal cysts caused by *S. pneumoniae* has been reported to date. Based on our knowledge, our patient is the first reported case of *S. pneumoniae* infection of a simple renal cyst in a previously simple renal cyst.

Simple cysts are usually asymptomatic, but they occasionally become symptomatic. In children, simple renal cysts may be discovered as a result of abdominal pain, urinary tract infection, or unknown fever [17, 18], but few cases with microbiological evidence of renal cyst infection have been reported. Chalkley [19] in 1943 recorded the first case of a child with an infected renal cyst who had dull pain in the right upper quadrant of her abdomen 3 weeks prior to admission and had pyuria on admission. She underwent a right nephrectomy, and the culture of fluid from the cyst yielded *Staphylococcus albus*. The mechanism by which the cyst becomes infected remains uncertain. The development from ascending infections of the lower urinary tract [20] or by hematogenous seeding from primary infected sites [21] or direct penetration, such as biopsy and operation [22], were presumptively considered. Patel [23] in 1978 reviewed 24 cases from the world literature and added 2 cases of their own. Out of these 2 cases where reports of the culture of the cyst content were available, 6 (30.1 %) had no organisms detected in the pus. In our case, a simple renal cyst had been recognized ten months before admission. Pneumonitis, otitis media and pneumococcal bacteremia were observed, but pyuria and bacteriuria were not found in urinalysis findings at the time of admission, and admission urine culture was also negative. There were no abnormal findings on ultrasonography at the time of admission, but ultrasonography on the day after admission showed a slightly thicker wall and increased internal brightness than that at admission. An enhanced CT of the abdomen performed on hospital day 21 showed an enhanced thick wall and perirenal inflammatory changes in the preexisting cyst in the right kidney. We diagnosed pneumococcal infection of a simple renal cyst, as we identified *S. pneumoniae* from the drainage. We could not examine whether the serotype of the strain detected from the pus was consistent with that identified in blood cultures during bacteremia because the culture of the drainage fluid from the renal cyst was negative. It was considered that a preceding invasive pneumococcal infection might have caused subsequent infection of the simple renal cyst, and the antibiotic treatment could treat her pneumonitis, otitis media and bacteremia, while the infected simple renal cyst remained and developed because of poor drug penetration into the renal cyst.

It was reported that a pathogen was successfully isolated in 81 % of renal cyst aspirate cultures [2]. Although the conventional gold standard method for the identification of bacteria is culture, caution is required because the identification rate by culture is decreased from samples collected after the use of antibacterial drugs. Additionally, culture-negative specimens make the diagnosis and rationalization of therapy difficult. It has been recently reported that a test using 16 S rDNA is useful for the proper use of antibacterial agents. O’Donnell et al. [24] retrospectively reviewed 16 S rRNA PCR results from 78 specimens in 60 patients, and de-escalation to a narrower spectrum agent upon receipt of positive 16 S rRNA PCR was possible for 5/24 (21 %) patients. They could estimate the total daily cost savings of €866 in patients with antibiotic de-escalation and of €472 in patients with discontinuation of antibiotics. Niimi [4] reported the novel “melting temperature (Tm) mapping method” for rapidly identifying the dominant bacteria in a clinical sample by using the 16 S rRNA gene. Because this method can identify unknown pathogenic bacteria within three hours of whole blood collection without using a culture test, it could be used particularly in cases where rapid testing is required or to detect dead bacteria after antibiotic treatment. However, due to the false positive results caused by contamination with environmental bacteria, the method must be simplified and mechanized to ensure that contamination does not occur in the inspection process. The medical cost of the Tm mapping method for one clinical specimen is estimated to be 10,000 Japanese yen (€77). In this case, the Tm mapping method was performed using drainage pus directly after puncture, and *S. pneumoniae* could be rapidly identified 4.5 hours after drainage. The de-escalation from TAZ/PIPC to ABPC based on the positive result of the Tm mapping method could save 2,187 Japanese yen per day. After subtracting the cost of Tm mapping, we estimated that the change in antibacterial drugs alone reduced the medical cost by 72,005 Japanese yen (€552) after the de-escalation.

The international consensus statement on the management of infected ADPKD in children was published recently [8], but so far, there is no solid evidence in the medical literature for the optimal management to treat an infected renal cyst. Surgical intervention is favored because of poor antibiotic penetration to the infected renal cyst. Ohkawa et al. [25] intramuscularly injected amikacin into 9 patients with simple renal cysts and 2 patients with infectious renal cysts and measured the concentration of antibiotics in the cyst fluid. In the case of uninfected simple renal cysts, the drug concentration of the cysts was at the detection limit, while in infectious renal cysts, it was less than half of that in the serum. Patterson [26] suggested percutaneous drainage combined with a two-week course of antibiotics for treatment. In our case, drug-susceptibility testing against *S. pneumoniae* demonstrated sensitivity to all first-line drugs, but initial medical treatment with the antimicrobial agent did not sufficiently improve the disease activity of the infected renal cyst because of poor drug penetration. Percutaneous drainage and subsequent 2-week antibiotic treatment were implemented and improved the condition of the patient. Transdermal suction or drainage may be necessary because infectious simple renal cysts do not provide adequate antimicrobial penetration.

In conclusion, we first reported a patient with a simple renal cyst infected with *S. pneumoniae*, as detected by the Tm mapping method, who showed negative culture of the drainage. Rapid identification of the microorganism allowed us to perform appropriate antimicrobial treatment and decrease the cost. The Tm mapping method, which has a high sensitivity, may be a useful antimicrobial stewardship tool for targeting antimicrobial therapy.

## Data Availability

The datasets supporting the conclusions of this article are included within the manuscript.

## Abbreviations

WBC: White blood cell
CRP: C-reactive protein
CT: Computed tomography
Tm: Melting temperature
CTX: Cefotaxime
VCM: Vancomycin
PCR: Polymerase chain reaction
ABPC: Ampicillin
TAZ/PIPC: Tazobactam-piperacillin
ADPKD: Autosomal dominant polycystic kidney disease
*E.coli*: *Escherichia coli*
*S. pneumoniae*: *Streptococcus pneumoniae*
